# Predictive Value of Ionized Calcium in Critically Ill Patients: An Analysis of a Large Clinical Database MIMIC II

**DOI:** 10.1371/journal.pone.0095204

**Published:** 2014-04-15

**Authors:** Zhongheng Zhang, Xiao Xu, Hongying Ni, Hongsheng Deng

**Affiliations:** Department of Critical Care Medicine, Jinhua Municipal Central Hospital, Jinhua Hospital of Zhejiang University, Zhejiang, P.R. China; D'or Institute of Research and Education, Brazil

## Abstract

**Background and Objective:**

ionized calcium (iCa) has been investigated for its association with mortality in intensive care unit (ICU) patients in many studies. However, these studies are small in sample size and the results are conflicting. The present study aimed to establish the association of iCa with mortality by using a large clinical database.

**Methods:**

Multiparameter Intelligent Monitoring in Intensive Care II (MIMIC II) database was used for analysis. Patients older than 15 years were eligible, and patients without iCa measured during their ICU stay were excluded. Demographic data and clinical characteristics were extracted and compared between survivors and non-survivors. iCa measure on ICU admission was defined as Ca_0_; Ca_max_ was the maximum iCa during ICU stay; Ca_min_ was the minimum value of iCa during the ICU stay; Ca_mean_ was the arithmetic mean iCa during ICU stay.

**Main results:**

A total of 15409 ICU admissions satisfied our inclusion criteria and were included in our analysis. The prevalence of hypocalcemia on ICU entry was 62.06%. Ca_0_ was significantly lower in non-survivors than in survivors (1.11±0.14 vs 1.13±0.10 mmol/l, p<0.001). In multivariate analysis, moderate hypocalcemia in Ca0 was significantly associated with increased risk of death (OR: 1.943; 95% CI: 1.340–2.817), and mild hypercalcemia was associated with lower mortality (OR: 0.553, 95% CI: **0.400–0.767**). While moderate and mild hypocalcemia in Ca_mean_ is associated with increased risk of death (OR: 1.153, 95% CI: 1.006–1.322 **and** OR: 2.520, 95% CI: 1.485–4.278), hypercalcemia in Ca_mean_ is not significantly associated with ICU mortality.

**Conclusion:**

The relationship between Ca_0_ and clinical outcome follows an “U” shaped curve with the nadir at the normal range, extending slightly to hypercalcemia. Mild hypercalcemia in Ca_0_ is protective, whereas moderate and mild hypocalcemia in Ca_mean_ is associated with increased risk of death.

## Introduction

Derangement in ionized calcium (iCa) is common in both surgical and medical patient requiring intensive care unit (ICU) admission. ICU patients are critically ill and multiple mechanisms underline the pathophysiological pathways of calcium derangement. Hypocalcemia is thought to be caused by the following mechanisms: i) increased fecal and/or urinary excretory Ca^2+^ losses in the presence of fixed dietary Ca2+ intake; ii) catecholamine-mediated translocation of plasma Ca2+ into tissues; and iii) reduced dietary Ca2+, often in association with vitamin D deficiency.[1] In critically illness, heart failure and hyperadrenergic states are the most commonly seen disorders that have been proven to be associated with calcium derangements.[2], [3]

Laboratory measurement of ionized calcium is readily available in most modern ICUs and thus the determination of its clinical significance has both prognostic and therapeutic values. For instance, if iCa is associated with clinical outcome, will therapeutic interventions aiming to restore calcium homeostasis be beneficial for ICU patients? Many preliminary investigations have been conducted to examine the prognostic value of iCa in critically ill patients. However, these studies are relatively small in sample size and their results were conflicting.[4]–[6] In the present study, we aimed to determine the association of iCa and clinical outcome by using a large clinical database named Multiparameter Intelligent Monitoring in Intensive Care II (MIMIC II).[7] We hypothesized that derangement in iCa was associated with altered ICU mortality.

## Method

### The Database

The MIMIC II (version 2.6) clinical database consisted of more than 30,000 ICU patients (medical, surgical, coronary care and neonatal) admitted to Beth Israel Deaconess Medical Center (Boston, MA) from 2001 to 2008. The establishment of the database was approved by the Institutional Review Boards of the Massachusetts Institute of Technology (Cambridge, MA) and Beth Israel Deaconess Medical Center (Boston, MA). Our access to the database was approved after completion of the NIH web-based training course named “Protecting Human Research Participants” by the author Z.Z. (certification number: 1132877). Informed consent was waived due to observational nature of the study. The study was approved by the ethics committee of Jinhua municipal central hospital. Data extraction was performed by using structure query language (SQL) with pgADmin PostgreSQL tools (version 1.12.3). MIMIC II was a relational database consisted of 38 tables. Data were extracted from the following tables: LABEVENTS, POE_MED, POE_ORDER, COMORBIDITY_SCORES, ICUSTAY_DETAIL.

### Study population and definitions

Patients older than 15 years were enrolled into the present analysis. Patients without iCa measured during their ICU stay were excluded. Furthermore, because renal replacement therapy (RRT) might have significant impact on serum iCa, sensitivity analysis was performed by excluding patients underwent RRT. Data on following information were extracted: age on ICU admission, sex, Elixhauser comorbidity score, type of ICU (including coronary care, medical, surgical, and cardiac surgery care units), day 1 sequential organ failure assessment (SOFA) and Simplified Acute Physiology Score (SAPS-1), time of ICU admission and discharge, date of death, all measurements of iCa during ICU stay. Comorbidities including hypertension, paralysis, chronic pulmonary disease, diabetes, renal failure, acquired immunodeficiency syndrome (AIDS), coagulopathy, obesity and weight loss were also abstracted. Types of ICU including coronary care unit (CCU), cardiac surgery care units (CSRU), medical intensive care unit (MICU) and surgical intensive care unit (SICU) were incorporated into analysis.

The primary endpoint in our study was the ICU mortality which was defined as death observed during ICU stay. iCa was analyzed by using potentiometric titration. There was no protocol specifying on which clinical condition should iCa be obtained and the measurement was determined totally by the treating physician. Because iCa was measured serially during ICU stay, we made several definitions for the ease of communication. iCa measure on ICU admission was defined as Ca_0_; Ca_max_ was the maximum iCa during ICU stay; Ca_min_ was the minimum value of iCa during the ICU stay; Ca_mean_ was the arithmetic mean iCa during ICU stay.

### Statistical analysis

Continuous variables were tested for normality by using Kolmogorov–Smirnov test. Data of normal distribution were expressed as mean±SD and compared using t test. Otherwise, Wilcoxon rank-sum test was used for comparison. Categorical variables were expressed as percentage and compared using Chi square test or Fisher's exact test as appropriate. ICU mortality was used as the study endpoint. To exclude confounding factors that may influence the association of iCa and mortality, logistic regression model was used to adjust for the odds ratios (OR). We built two models separately for Ca_0_ and Ca_mean_ during ICU stay. The full model included all variables listed in Table 1.[8] Covariate selection was performed by using stepwise forward selection and backward elimination technique, with Ca_0_ and Ca_mean_ remaining in the model. The significance level for selection was predefined as 0.15 and that for elimination was 0.2. After this step the main effect model was built. Lowess smooth technique was used to examine the relationship between iCa and mortality in logit.[9] To facilitate clinical interpretation of our results and to meet the interests of subject-matter audience, we planned to use linear spline function for model building.[10] The knots were chosen according to conventional classification of iCa ranges: relative to the normal range of 1.15–1.25 mmol/L, we defined hypocalcemia as mild, moderate and severe as 0.9–1.15, 0.8–0.9 and <0.8 mmol/L, respectively. Hypercalcemia was divided into mild, moderate and severe as 1.25–1.35, 1.35–1.45 and >1.45 mmol/L, respectively.[11], [12] Potential multicollinearity between covariates in the model were quantified by using variance inflation factor (VIF) which provided an index that measures how much the variance of an estimated regression coefficient is increased because of collinearity.[13] As a common rule of thumb, a VIF>5 was considered for the existence of multicollinearity. Furthermore, iCa was categorized into intervals and incorporated into regression models as design variable. Design variable, also known as dummy variable, is one that takes the value of 0 or 1 to indicate the presence or absence of some categorical effect that is expected to shift the outcome. It is frequent used for categorical variables with more than two categories. Normal range between 1.15 and 1.25 mmol/l was used as reference and ORs were reported for other intervals. Receiver operating characteristic curve (ROC) was depicted to show the diagnostic performance of fitted logistic regression models.

**Table 1:** Characteristics between intensive care unit survivors and non-survivors.

Characteristics	Survivors (n = 13754)	Non-survivors (n = 1655)	p
Age (years)	64.0±19.9	69.0±16.2	<0.001
Sex (male, %)	8205 (59.7%)	921 (55.7%)	0.002
SAPSI on admission	15.6±4.9	19.7±5.6	<0.001
SOFA on admission	7.2±3.7	10.1±4.6	<0.001
Comorbidity (n, %)			
Congestive heart failure	2731 (19.88%)	459 (27.77%)	<0.001
Paralysis	198 (1.44%)	28 (1.69%)	0.420
Renal failure	773 (5.63%)	134 (8.11%)	<0.001
Uncomplicated diabetes	2766 (20.13%)	291 (17.60)	0.015
Complicated diabetes	738 (5.37%)	87 (5.26%)	0.853
Coagulopathy	815 (6.19%)	153 (9.26%)	<0.001
AIDS	78 (0.57%)	12 (0.73%)	0.425
Chronic pulmonary disease	2254 (16.41%)	285 (17.24%)	0.387
Obesity	281 (2.05%)	14 (0.85%)	<0.001
Weight loss	427 (3.11%)	67 (4.05%)	0.039
Types of care unit (n, %)			
CCU	2019 (14.68%)	369 (22.30%)	<0.001
CSRU	6919 (50.31%)	400 (24.17%)	<0.001
MICU	4080 (29.66%)	799 (48.28%)	<0.001
SICU	736 (5.35%)	87 (5.26%)	0.872
Ionized calcium (mmol/l)			
Ca_0_	1.13±0.10	1.11±0.14	<0.001
Ca_mean_	1.14±0.19	1.11±0.15	<0.001
Ca_max_	1.26±1.86	1.27±2.05	0.577
Ca_min_	1.04±0.12	1.00±0.15	<0.001

Abbreviations: CCU, coronary care unit; CSRU, cardiac surgery care units; MICU, medical intensive care unit; SICU, surgical intensive care unit; SOFA, sequential organ failure assessment; SAPS, Simplified Acute Physiology Score.

All statistical analyses were performed using the software STATA 11.2 (College Station, Texas 77845 USA). Two-tailed p<0.05 was considered to be statistically significant.

## Results

A total of 15409 ICU admissions satisfied our inclusion criteria and were included in our analysis. Nine thousand five hundred and sixty-three (62.1%) patients had hypocalcemia on ICU admission, in which there were 105 patients with severe hypocalcemia, 265 with moderate hypocalcemia and 9193 with mild hypocalcemia. One thousand two hundred and seven (7.8%) patients had hypercalcemia on admission, including 865, 226 and 116 patients in respective mild, moderate and severe hypercalcemia groups. There were 13754 survivors and 1655 non-survivors during ICU stay, with the ICU mortality rate of 10.7% (Table 1). Non-survivors were significantly older than survivors (69.0±16.2 vs 64.0±19.9 years, p<0.001), and there were more male patients in survivors than that in non-survivors (59.7% vs 55.7%, p = 0.002). As expected, SOFA and SAPS-1 scores were both significantly higher in non-survivors than in survivors (10.1±4.6 vs 7.2±3.7, p<0.001; 19.7±5.6 vs 15.6±4.9, p<0.001). Comorbidities were significantly different between survivors and non-survivors. There were more patients with congestive heart failure (27.77% vs 19.88%; p<0.001), renal failure (8.11% vs 5.63%, p<0.001), coagulopathy (9.26% vs 6.19%, p<0.001), and weight loss (4.05% vs 3.11%, p = 0.039) in non-survivors than in survivors. On the contrary, there were less patients with uncomplicated diabetes (17.60% vs 20.13%, p = 0.015) and obesity (0.85% vs 2.05%, p<0.001) in non-survivors than in survivors. Patients admitted to CCU (22.30% vs 14.68%, p<0.001) and MICU (48.28% vs 29.66%, p<0.001) were more likely to die, whereas patients in CSRU (24.17% vs 50.31%, p<0.001) were less likely to die. Ca_0_ was significantly lower in non-survivors than in survivors (1.11±0.14 vs 1.13±0.10 mmol/l, p<0.001). Ca_mean_ was also significantly lower in non-survivors than in survivors (1.11±0.15 vs 1.14±0.19 mmol/l, p<0.001). Ca_max_ was not statistically different between survivors and non-survivors.

**Figure 1:**
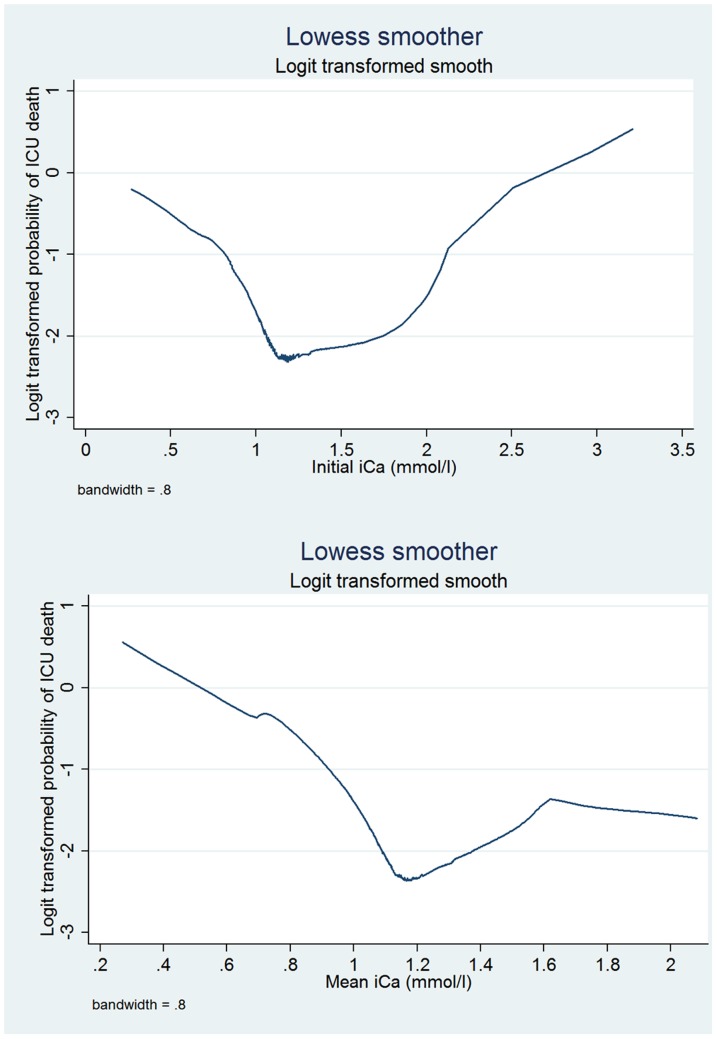
Relationship between initial iCa (upper panel) and logit transformed mortality by using Lowess smoothing technique. The figure shows that the relationships are non-linear. The lowest mortality rate occurs within the normal range of iCa (1.15–1.25 mmol/l). For the initial iCa, the “U” shape curve indicates that both hyper-and hypocalcemia are associated with equally increased risk of death. However, for the mean iCa, hypocalcemia was associated with more death than hypercalcemia.

Table 2 displays the main effect model built by using stepwise forward selection and backward elimination technique for Ca_0_ and Ca_mean_. Both models contained the same variables, including age, sex, SAPS-1, SOFA, congestive heart failure, paralysis, CSRU, uncomplicated diabetes, MICU and obesity were remained in the model. Figure 1 shows the relationship between iCa and logit transformed probability of death. The result showed that both Ca_0_ and Ca_mean_ were non-linear in the model. We used linear spline function to explore the non-linear function. Furthermore, we divided iCa into categories and transformed it into design variable, with the normal range of 1.15–1.25 mmol/l as the reference group.

**Table 2:** Main effect model derived using stepwise forward selection and backward elimination technique.

Model 1				Model 2			
Variable	Odds ratio	95% CI	p	Variable	Odds ratio	95% CI	p
Ca_0_	0.317	0.187–0.540	<0.001	Ca_mean_	0.288	0.140–0.591	0.001
Age	1.008	1.005–1.011	<0.001	Age	1.008	1.005–1.011	<0.001
Sex	1.104	0.979–1.245	0.107	Sex	1.101	0.977–1.243	0.116
SAPS-I	1.110	1.093–1.127	<0.001	SAPS-I	1.109	1.093–1.126	<0.001
SOFA	1.126	1.104–1.148	<0.001	SOFA	1.126	1.105–1.148	<0.001
Congestive heart failure	1.147	0.998–1.317	0.053	Congestive heart failure	1.145	0.997–1.315	0.055
paralysis	1.399	0.914–2.142	0.122	paralysis	1.402	0.916–2.145	0.120
CSRU	0.277	0.236–0.325	<0.001	CSRU	0.280	0.238–0.329	<0.001
Uncomplicated diabetes	0.798	0.683–0.933	0.005	Uncomplicated diabetes	0.799	0.684–0.934	0.005
Complicated diabetes	0.791	0.597–1.047	0.101	Complicated diabetes	0.792	0.598–1.049	0.104
MICU	1.106	0.957–1.277	0.171	MICU	1.111	0.962–1.283	0.153
Obesity	0.624	0.352–1.104	0.105	Obesity	0.615	0.347–1.091	0.096

Note: The variance inflation factor (VIF) for model 1 is 1/(1-R^2^) = 1/(1−0.1661) = 1.199; and for model 2 is 1/(1-R^2^) = 1/(1−0.1657) = 1.199.

Abbreviations: SOFA, sequential organ failure assessment; SAPS, Simplified Acute Physiology Score; CSRU, cardiac surgery care units; MICU, medical intensive care unit.

Table 3 shows the different adjusted odds ratios of iCa in different intervals. For Ca_0_<1.15, the odds ratios were less than 1, suggesting that the probability of ICU death decreased with increasing Ca_0_. The OR was 0.0006 between 1.25 and 1.35, indicating that mild hypercalcemia on ICU admission was associated with decreasing mortality. Severe hypercalcemia (>1.35 mmol/l) was associated with increased risk of death (OR: 3515.89 and 6.814 for each unit increase in iCa for the intervals 1.35–1.45 and >1.45, respectively). For Ca_mean_ in the range of 0.9–1.15 mmol/l, the OR was 0.016 (95% CI: 0.004–0.0581) for each unit increase in Ca_mean_. Multicolinearity among covariates could be excluded in the model as reflected by a VIF of 1.204.

**Table 3:** Multivariable logistic regression categorizing calcium values into intervals by using linear spline function.

Model 1^†^				Model 2^‡^			
Variables	Odds ratio	95% CI	p	Variables	Odds ratio	95% CI	p
Severe hypocalcemia (<0.8)	0.853	0.016–44.13	0.937	Severe hypocalcemia (<0.8)	0.021	2.48×10^−5^–17.11	0.258
Moderate hypocalcemia (0.8–0.9)	0.078	0.0002–27.4	0.393	Moderate hypocalcemia (0.8–0.9)	243.59	0.023–2.56	0.245
Mild hypocalcemia (0.9–1.15)	0.069	0.023–0.209	<0.001	Mild hypocalcemia (0.9–1.15)	0.0161	0.004–0.0581	<0.001
Normal (1.15–1.25)	7.358	0.619–87.43	0.114	Normal (1.15–1.25)	3.188	0.175–58.04	0.434
Mild hypercalcemia (1.25–1.35)	0.0006	1.47×10^−6^−0.272	0.017	Mild hypercalcemia (1.25–1.35)	0.127	9.96×10^−5^–163.06	0.572
Moderate hypercalcemia (1.35–1.45)	3515.891	0.459–2.69×10^7^	0.074	Moderate hypercalcemia (1.35–1.45)	8696.2	0.348–2.17×10^8^	0.079
Severe hypercalcemia (>1.45)	6.814	0.84–55.23	0.072	Severe hypercalcemia (>1.45)	0.856	0.494–1.485	0.581
Age	1.009	1.006–1.011	<0.001	Age	1.009	1.006–1.011	<0.001
Sex	1.096	0.971–1.237	0.137	Sex	1.097	0.972–1.238	0.133
SAPS-1	1.107	1.091–1.124	<0.001	SAPS-1	1.107	1.090–1.124	<0.001
SOFA	1.124	1.103–1.146	<0.001	SOFA	1.123	1.101–1.145	<0.001
Congestive heart failure	1.145	0.997–1.136	0.056	Congestive heart failure	1.151	1.002–1.322	0.047
Paralysis	1.387	0.904–2.129	0.134	Paralysis	1.402	0.915–2.149	0.121
Uncomplicated diabetes	0.802	0.686–0.938	0.006	Uncomplicated diabetes	0.809	0.692–0.946	0.008
Complicated diabetes	0.797	0.602–1.055	0.113	Complicated diabetes	0.804	0.607–1.065	0.128
Obesity	0.620	0.349–1.101	0.103	Obesity	0.626	0.352–1.113	0.111
MICU	1.101	0.953–1.273	0.191	MICU	1.096	0.948–1.266	0.217
CSRU	0.278	0.236–0.326	<0.001	CSRU	0.294	0.250–0.345	<0.001

Note: ^†^model 1 contains initial calcium (Ca_0_) and the variance inflation factor (VIF) was 1.204; ^‡^model 2 contains mean calcium (Ca_mean_) and the VIF was 1.204.

Abbreviations: SOFA, sequential organ failure assessment; SAPS, Simplified Acute Physiology Score; CSRU, cardiac surgery care units; MICU, medical intensive care unit.

**Figure 2:**
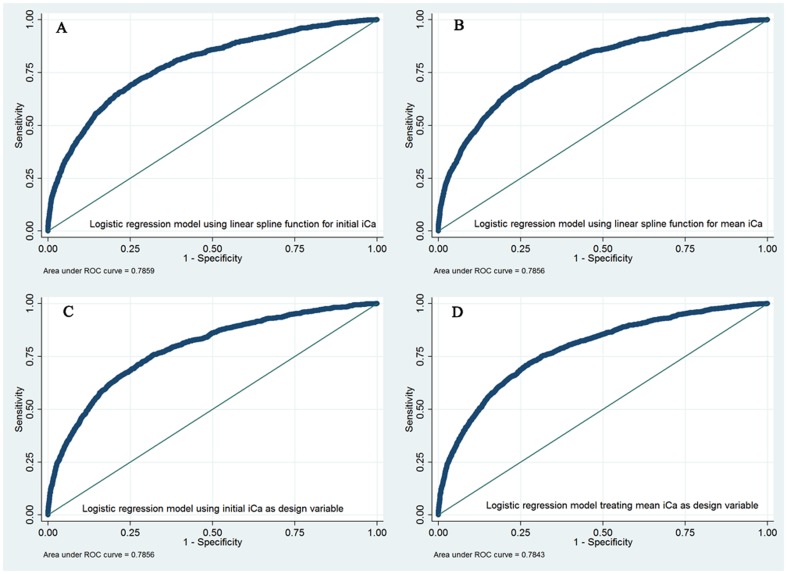
Receiver operating characteristic curve (ROC) shows that the areas under ROC are approximately 0.78 for the four fitted models.

Table 4 shows the multivariable logistic regression model by incorporating iCa as design variable. The result showed that moderate hypocalcemia was significantly associated with increased risk of death (OR: 1.943; 95% CI: 1.340–2.817); and mild hypercalcemia was associated with lower mortality (OR: 0.553, 95% CI: 0.400–0.767). On the other hand, mild and severe hypocalcemia, moderate and severe hypercalcemia measured on ICU entry were not associated with altered ICU mortality. Ca_mean_ was also investigated for its association with mortality in the multivariate model. The results showed that both mild and moderate hypocalcemia were associated with significantly increased risk of death (OR: 1.153, 95% CI: 1.006–1.322; OR: 2.520, 95% CI: 1.485–4.278). Hypercalcemia was associated with increased risk of death, but statistical significance was not reached. Multicolinearity among covariates could be excluded in the models (VIF<5). Figure 2 displays the examination of diagnostic performance of fitted model by using ROC. The result showed that the diagnostic performances were moderately good with areas under ROC of around 0.78. A total of 139 patients had undergone RRT. Sensitivity analysis by excluding these patients did not significantly change the result (data not shown).

**Table 4:** Multivariable logistic regression categorizing calcium values into design variables.

Model 1				Model 2			
Variables	Odds ratio	95% CI	p	Variables	Odds ratio	95% CI	p
Referent group with Ca_0_ 1.15–1.25	1			Referent group with Ca_mean_ 1.15–1.25	1		
Severe hypocalcemia (<0.8)	1.712	0.971–3.019	0.063	Severe hypocalcemia (<0.8)	1.332	0.478–3.716	0.583
Moderate hypocalcemia (0.8–0.9)	1.943	1.340–2.817	<0.001	Moderate hypocalcemia (0.8–0.9)	2.520	1.485–4.278	0.001
Mild hypocalcemia (0.9–1.15)	1.014	0.888–1.159	0.832	Mild hypocalcemia (0.9–1.15)	1.153	1.006–1.322	0.041
Mild hypercalcemia (1.25–1.35)	0.553	**0.400–0.767**	<0.001	Mild hypercalcemia (1.25–1.35)	0.872	0.597–1.274	0.48
Moderate hypercalcemia (1.35–1.45)	0.976	0.588–1.**619**	0.924	Moderate hypercalcemia (1.35–1.45)	1.439	0.782–2.647	0.243
Severe hypercalcemia (>1.45)	1.407	0.760–2.605	0.277	Severe hypercalcemia (>1.45)	1.975	0.888–4.390	0.095
Age	1.008	1.005–1.011	<0.001	Age	1.008	1.005–1.011	<0.001
Sex	1.100	0.975–1.241	0.121	Sex	1.109	0.983–1.251	0.094
SAPS-1	1.110	1.094–1.127	<0.001	SAPS-1	1.110	1.093–1.127	<0.001
SOFA	1.126	1.105–1.148	<0.001	SOFA	1.126	1.104–1.148	<0.001
Congestive heart failure	1.145	0.997–1.316	0.055	Congestive heart failure	1.143	0.996–1.313	0.058
Paralysis	1.384	0.903–2.120	0.135	Paralysis	1.380	0.901–2.115	0.139
Uncomplicated diabetes	0.796	0.681–0.930	0.004	Uncomplicated diabetes	0.802	0.687–0.938	0.006
Complicated diabetes	0.789	0.596–1.045	0.098	Complicated diabetes	0.795	0.600–1.053	0.109
Obesity	0.614	0.345–1.091	0.096	Obesity	0.622	0.351–1.102	0.104
MICU	1.106	0.958–1.278	0.17	MICU	1.104	0.956–1.276	0.178
CSRU	0.276	0.235–0.324	<0.001	CSRU	0.279	0.237–0.327	<0.001

Note: ^†^model 1 contains initial calcium (Ca_0_) and the variance inflation factor (VIF) was 1.201; ^‡^model 2 contains mean calcium (Ca_mean_) and the VIF was 1.199.

Abbreviations: SOFA, sequential organ failure assessment; SAPS, Simplified Acute Physiology Score; CSRU, cardiac surgery care units; MICU, medical intensive care unit.

## Discussion

The study shows that both Ca_0_ and Ca_mean_ are associated with altered ICU mortality in unselected critically ill patients, but in a complex form. To the best of our knowledge, this is the largest study to establish the linkage between derangement in iCa and morality in mixed ICU patients. The finding that mild hypercalcemia provided protective effect on mortality suggests that calcium supplementation may potentially benefit critically ill patients.

One advantage of the study is the use of MIMIC II clinical database.[14] This database comprises high resolution clinical information of more than 30000 ICU admissions. The data comprising MIMIC-II was collected at the Beth Israel Deaconess Medical Center in Boston over an eight-year span from 2001 to 2008. Although there are other clinical databases available for research in critical care medicine,[15], [16] MIMIC II is one of the largest clinical database that can provide high resolution clinical information. Most importantly, this database is freely available to public users. The advantage of using such existing database is that it represents the “real world” setting in which no strict study protocol has been performed in collecting data. In contrast, interventional trials have been criticized for its strict inclusion and exclusion criteria, its performance in specialized centers, and its management does not represent usual care.[17]

A recent study conducted by Steele T and colleagues failed to identify the association of hypocalcemia with morality in a cohort of heterogeneous ICU patients (p = 0.33).[18] This study is small sample sized including only 1000 ICU admissions, which significantly compromises the statistical power of the study. Furthermore, the 28-day mortality rate is significantly lower in Steele's study than that in our study. Most probably, the negative impact of hypocalcemia is only present in more severely ill patients. In another study by Egi M and colleagues,[12] they found that iCa on ICU admission was not significantly different between survivors and non-survivors, and only extreme hypo- or hyper-calcemia was independent predictors of mortality. This is in contrast to our findings that both severe hypocalcemia and hypercalcemia are not independent predictors of outcome. However, Egi's study found that incident (occurring once during ICU stay) hypocalcemia and hypercalcemia were significantly associated with worse clinical outcome. It is probably that there is no consensus on the management of iCa derangement in ICU and the protocol may vary across institutions. In Egi's study, intravenous calcium supplementation is given only for severe hypocalcemia associated with bleeding; but the protocol is not explicitly reported in the MIMIC II database. Calcium supplementation has been investigated in both animal and clinical studies for its effect on mortality, showing that calcium supplementation will have negative impact on clinical outcomes.[19] Since there is not specific calcium management protocol in these studies, such confounding effect cannot be excluded. Consistently with our study, Choi YC and colleagues [20] identified a strong association between initial hypocalcemia and mortality in 255 consecutive trauma patients, and this association remained after adjustment of important confounders. Also, another two studies, one conducted in emergency department and the other in ICU, consistently reported a strong association between on-admission hypocalcemia and mortality.[21], [22] In the later study, Hästbacka J and colleagues excluded patients who had received calcium supplementation to exclude the impact of the intervention on serum iCa.

Hypocalcemia is prevalent in ICU patients, as reported in our study that 62% patients had hypocalcemia on ICU admission. The prevalence is similar to that reported by Iqbal M and colleagues.[23] Calcium homeostasis is regulated by the vitamin D-parathyroid-calcium axis and critical illness has shown to be associated with dysfunction of this axis. Proposed mechanisms include impairment of parathyroid hormone by pro-inflammatory cytokines, catecholamine excess in ICU patients, end organ resistance to parathyroid hormone, inhibition of parathyroid hormone secretion and cellular redistribution of iCa.[24], [25] A recent study conducted by Nair P and colleagues [26] showed that vitamin D insufficiency or deficiency were prevalent among ICU patients (78%) and the level did not recover during treatment. This prevalent hypovitaminosis D explains the high incidence of hypocalcecmia as reported in our study and many others. Although hypovitaminosis D was not reported to be associated with higher mortality in Nair's study, it was associated with worse disease severity and fewer hospital-free days. Probably, that study was under-powered to detect a difference of mortality due to limited sample size (100 subjects).

Hypercalcemia was investigated in our study and the result showed that mild hypercalcemia was associated with reduction in mortality risk, and the relationship was only valid for Ca_0_ rather than Ca_mean_. One plausible explanation is that Ca_mean_ can be influenced by calcium supplementation during ICU stay. If hypocalcemia is a marker of disease severity, elevation of iCa by supplementation may not necessarily translate to clinical benefit. The protective effect of mild hypercalcemia is a unique finding in our study and has never been reported in the literature. In previous mentioned study by Egi M and colleagues, incident mild hypercalcemia (the same reference range) was associated with increased risk of death. Another two small studies failed to identify statistically significant association between hypercalcemia and mortality.[27], [28] Severe hypercalcemia is thought to be associated with increase in mortality risk. Great majority of patients with severe hypercalcemia is attributable to either primary hyperparathyroidism or malignancy.[29] These comorbidities *per se* are associated with increased mortality. On the other hand, severe hypercalcemia is a well-known risk factor for acute kidney injury (AKI) in critically ill patients, and the occurrence of AKI has been associated with increased mortality.[30], [31] However, severe hypercalcemia in neither Ca_0_ nor Ca_mean_ was associated with increase in mortality risk in the present study. Actually, severe hypercalcemia is rarely seen in ICU patients, accounting for only 0.75% in our study. The limited sample size may significantly compromise the statistical power. As shown in table 4, severe hypercalcemia is associated with 1.4-fold increase in the risk of death, but statistical significance is not reached. This is most probably attributable to the limited sample size in severe hypercalcemia group. However, based on current evidence, the clinical significance of hypercalcemia cannot be determined and further investigations are needed.

There are several limitations need to be acknowledged. First, the study is retrospective in nature and bears potential limitations of such design. For instance, patients without iCa measured during ICU stay were excluded from the analysis, this may cause bias that the included cohort cannot represent the whole study population. However, included and excluded cohorts are similar in many clinical characteristics (data not shown), making our cohort representative of the target population. Second, ICU patients were heterogeneous including medical, surgical and cardiac surgical patients, whether narrowing study population will improve the prognostic value of iCa for mortality requires further investigations. Third, although every effort has been made to adjust for the confounding factors by using multivariate analysis, other unknown factors may still exist to confound the prognostic value of iCa. This may partly explain the disparity of the results between our study and others'. Finally, we used ICU mortality instead of the more commonly used ones such as 28-day and 90-day mortality as the study endpoint. This is because data are not directly available in the MIMIC-2 database after ICU discharge. Therefore, if we use 28-day or 90-day mortality, many patients discharged from ICU or hospital before the certain time period will be regarded as censored, and this will result in too many censored data.

## Conclusion

In aggregate, by the analysis of a large clinical database, our study shows that both hypocalcemia and hypercalcemia is associated with altered mortality, but in a complex form. Interestingly, mild hypercalcemia on ICU admission is found to be protective and is associated with reduction in mortality risk.
